# ORF3 Depletion Attenuates SADS‐CoV Virulence and Retains Partial Maternally Mediated Protection in Suckling Mice

**DOI:** 10.1155/tbed/6713584

**Published:** 2026-10-01

**Authors:** Nian-Qi Zhang, Wei Huang, Zhong-Li Zhang, Pan-Pan Zhao, Zhao-Yi Xu, Nian-Jin Zhang, Zhi-Gang Xie, Yong-Le Yang, Feng Cong, Yao-Wei Huang, Bin Wang

**Affiliations:** ^1^ State Key Laboratory of Animal Disease Control and Prevention Guangdong Laboratory for Lingnan Modern Agriculture College of Veterinary Medicine South China Agricultural University Guangzhou China scau.edu.cn; ^2^ Xianghu Laboratory Hangzhou China; ^3^ College of Animal Science and Technology Zhongkai University of Agriculture and Engineering Guangzhou China zhku.edu.cn

**Keywords:** live attenuated vaccine (LAV), maternal protection, ORF3, swine acute diarrhea syndrome coronavirus (SADS-CoV), virulence

## Abstract

Swine acute diarrhea syndrome coronavirus (SADS‐CoV) is an emerging enteric coronavirus that causes severe diarrhea and high mortality in neonatal piglets. Its broad host range, tropism for human‐origin cells, and limited vaccine availability underscore the importance of defining viral determinants of pathogenicity. The accessory protein ORF3 has been linked to CoV virulence, but its role in SADS‐CoV infection in vivo and its impact on maternally transferred protection have not been fully established. Here, we used reverse genetics to generate rSADS‐ΔORF3/GFP, a recombinant virus with ORF3 replaced by GFP, and compared it with parental rSADS‐CoV in suckling BALB/c mice. ORF3 depletion markedly attenuated disease and reduced viral RNA burdens across tissues relative to parental rSADS‐CoV. Because maternally derived immunity is critical to protection against swine enteric CoV infection in newborn animals, we further evaluated whether rSADS‐ΔORF3/GFP, as a potential live attenuated vaccine (LAV), could elicit maternal protection transferred from inoculated dams to their offspring. Compared with parental rSADS‐CoV, rSADS‐ΔORF3/GFP induced lower maternal neutralizing antibody titers and provided less robust protection in challenged offspring. Nevertheless, offspring from rSADS‐ΔORF3/GFP‐inoculated dams showed improved survival, weight gain, milder intestinal pathology, and lower tissue viral RNA burdens than offspring from DMEM‐inoculated dams, indicating retained protective immunogenicity. Together, these findings support a contribution of ORF3 to SADS‐CoV virulence and demonstrate that ORF3 depletion yields an attenuated virus that retains partial maternally mediated protective efficacy. The residual immunogenicity of rSADS‐ΔORF3/GFP, combined with its reduced virulence, supports its further evaluation as a LAV candidate.

## 1. Introduction

Swine acute diarrhea syndrome coronavirus (SADS‐CoV) is a bat‐related enteric alphacoronavirus that causes vomiting, dehydration, and severe diarrheal disease in newborn piglets [1, 2]. The disease was recognized after outbreaks in Guangdong, China, in 2016–2017 and has since been reported sporadically in several Chinese provinces, including Jiangxi, Fujian, and Guangxi [2–6]. More recent detection of SADS‐CoV RNA in Vietnamese pig‐farm samples, together with serological evidence of exposure in Korean swine herds, suggests that the virus may have spread beyond the regions in which it was first detected [7, 8]. SADS‐CoV contains an ~27‐kb positive‐sense RNA genome that encodes the CoV replicase, four structural proteins, and three accessory proteins, including ORF3 (also referred to as NS3), NS7a, and NS7b [9, 10].

Beyond its economic impact on the swine industry, SADS‐CoV has attracted attention because of its broad host range and cellular tropism. The virus can replicate in cells from a range of species, including human cell lines and primary human lung and intestinal cells, indicating a potential capacity to cross species barriers [11–13]. Experimental infection studies further showed that SADS‐CoV can establish productive infection in neonatal and suckling mice, causing severe disease in an age‐dependent manner [14]. In this model, the virus disseminates to multiple tissues, including the intestine and brain, and infection is accompanied by histopathological injury and inflammatory responses [15].

Among the accessory proteins encoded by SADS‐CoV, ORF3 represents a plausible determinant of viral pathogenicity. Coronavirus ORF3‐like proteins are multifunctional regulators that participate in viral replication, host membrane remodeling, immune evasion, and disease progression [16]. Many ORF3 proteins, particularly SARS‐CoV‐2 ORF3a and PEDV ORF3, possess viroporin‐like properties and modulate host cellular homeostasis through ion‐channel activity and membrane‐associated processes [17, 18]. At the cellular level, ORF3‐like proteins have been shown to interfere with endolysosomal trafficking, lysosomal function, and autophagy‐related pathways, thereby facilitating viral immune escape and efficient virus release [19–21]. Recent studies have demonstrated that SARS‐CoV‐2 ORF3a disrupts Rab7‐dependent lysosomal trafficking and impairs lysosomal function, resulting in altered cellular homeostasis and enhanced viral egress [19, 21]. In addition, ORF3a promotes inflammatory responses by activating the NLRP3 inflammasome and contributing to infection‐associated inflammation [22].

Beyond their effects on cellular organelles, ORF3‐like proteins are increasingly recognized as important modulators of antiviral immunity. SARS‐CoV and SARS‐CoV‐2 ORF3a proteins regulate innate immune activation, whereas PEDV ORF3 has been reported to suppress type‐I interferon signaling through interference with the RIG‐I‐like receptor (RLR) pathway, leading to reduced induction of IFN‐β and interferon‐stimulated genes (*ISGs*) [18, 22, 23]. Genetic studies further support the contribution of ORF3‐related accessory genes to coronavirus fitness and virulence, as deletion of ORF3‐associated regions in TGEV and removal of accessory genes 3a or 3b in IBV result in attenuation or altered pathogenicity in vivo [24–26]. Collectively, these findings establish coronavirus ORF3‐like proteins as context‐dependent determinants of viral replication, immune evasion, host adaptation, and pathogenicity. However, whether SADS‐CoV ORF3 similarly regulates viral pathogenicity and infection‐induced protective immunity remains incompletely understood, necessitating an in vivo model capable of evaluating both viral fitness and host immune protection.

Although neonatal piglets remain the natural host and the most relevant model for validating SADS‐CoV pathogenicity, piglet challenge experiments are costly, technically demanding, and poorly suited to rapid comparison of multiple recombinant viruses [3]. Suckling mice therefore provide a tractable, complementary system for the early in vivo assessment of SADS‐CoV virulence. Previous studies showed that neonatal BALB/c and C57BL/6J mice, especially 2‐day‐old mice, are susceptible to SADS‐CoV infection, exhibiting age‐dependent mortality, efficient viral replication, tissue dissemination, and histopathological injury [14, 15]. Therefore, we used suckling BALB/c mice to evaluate whether ORF3 contributes to SADS‐CoV virulence and infection‐induced maternally mediated protection.

Although SADS‐CoV ORF3 has been implicated in pathogenicity in piglets [27], its relationship with infection‐induced maternal antibody responses and protection of offspring remains incompletely understood. Moreover, in porcine neonatal CoV disease, the clinical outcome is shaped not only by viral virulence but also by maternally derived passive protection [28, 29]. Studies of PEDV have shown that passive lactogenic immunity delivered through colostrum and milk is a major determinant of protection in neonatal piglets, with virus‐specific IgA, IgG, and neutralizing antibodies in colostrum, milk, and offspring associated with protection after challenge [29, 30]. Thus, defining the role of ORF3 in SADS‐CoV infection requires assessing both viral pathogenicity and the maternally mediated protection induced by infection.

Here, we tested the hypothesis that SADS‐CoV ORF3 functions as a virulence factor whose deletion attenuates disease but may concomitantly reduce the immunogenicity required for maternally mediated protection, a tradeoff with direct implications for live attenuated vaccine (LAV) development. Using reverse genetics, we generated the ORF3‐deficient recombinant virus rSADS‐ΔORF3/GFP and compared it with the parental rSADS‐CoV in suckling BALB/c mice. ORF3 depletion attenuated disease and reduced multitissue viral burden but induced lower neutralizing antibody responses in dams and conferred weaker protection to offspring after challenge. These findings identify ORF3 as a determinant of SADS‐CoV virulence and maternally mediated protective immunity.

## 2. Materials and Methods

### 2.1. Cells and Virus

Vero and ST cells were obtained from the American Type Culture Collection (ATCC). Cells were cultured in Dulbecco’s modified Eagle medium (DMEM; Gibco, Cat. Number 11,965,092) supplemented with 10% fetal bovine serum, 100 units/mL penicillin, and 100 μg/mL streptomycin. Cells were incubated at 37°C with 5% CO_2_ under humidified conditions. The SADS‐CoV isolate CH/GD‐01/2017 at passage 10 (p10) was used in this study. Mycoplasma testing was performed at regular intervals, and all lines remained negative.

Antibodies and reagents. A polyclonal antibody against SADS‐CoV N (1:1000) was maintained in our laboratory. An anti‐β‐actin antibody was used at 1:1000 dilution (CST, Cat. Number 13E5). Alexa Fluor 568‐conjugated goat antimouse IgG (H + L) cross‐adsorbed secondary antibody (Thermo Fisher Scientific, Cat. Number A‐11004) and Alexa Fluor 488‐conjugated goat antirabbit IgG (H + L) cross‐adsorbed secondary antibody (Thermo Fisher Scientific, Cat. Number A‐11029) was used at 1:1000 dilution. DAPI (Cat. Number C0065, 1:1000) was purchased from Solarbio. The protein molecular weight marker used for western blotting was purchased from EpiZyme Biotechnology Co., Ltd. (Cat. Number WJ107L).

### 2.2. Ethics Statement

All animal procedures were reviewed and approved by the Institutional Animal Care and Use Committee of the Guangdong Laboratory Animals Monitoring Institute under Protocol IACUC2021167.

### 2.3. Mouse Infection

Wild‐type pregnant BALB/c mice were purchased from Guangzhou Ruige Biological Technology Co., Ltd. and housed under specific‐pathogen‐free conditions in an ABSL‐2 facility. Mice were maintained on a 12‐h light/dark cycle with free access to food and water. Suckling pups remained with their biological dams throughout the study.

For infection experiments, 2‐day‐old suckling mice were allocated to mock‐infected, rSADS‐CoV‐infected, or rSADS‐ΔORF3/GFP‐infected groups according to litter availability. To minimize litter‐associated bias, pups from different dams were distributed as evenly as possible across groups. Pups were orally inoculated with 1 × 10^6^ TCID_50_ of rSADS‐CoV or rSADS‐ΔORF3/GFP, and mock‐infected controls received the same volume of DMEM. Individual suckling mice were considered the experimental units.

Two independent cohorts were used. In the survival cohort, 12 pups per group were monitored daily for clinical signs, body‐weight change, and survival for 14 days after infection. Deaths and euthanasia after reaching humane endpoints were recorded as events for the survival analysis. In the tissue‐collection cohort, 18 pups per group were used for pathological and virological analyses. Six pups per group were euthanized at 3, 6, and 10 days postinfection. At each sampling point, pups were euthanized by carbon dioxide inhalation according to the approved IACUC protocol, and death was confirmed before tissue collection. The heart, liver, spleen, lung, kidney, stomach, small intestine, large intestine, and brain were collected for subsequent analyses.

Sample size was determined on the basis of previous SADS‐CoV suckling‐mouse infection studies, the requirement for tissue collection at multiple time points, and the principle of minimizing animal use. No pups were excluded before group allocation. Pups were excluded from scheduled tissue collection only if they died before the planned sampling point or if the collected samples were unsuitable for analysis. Animals were monitored daily for activity, suckling behavior, dehydration, and general condition. Humane endpoints included severe weakness, inability to suckle, persistent recumbency, severe dehydration, or a moribund state. Animals that reached humane endpoints were euthanized immediately by carbon dioxide inhalation.

### 2.4. Maternal Protection Assay

For maternal‐protection experiments, female BALB/c mice were assigned, according to animal availability, to three treatment groups: DMEM, rSADS‐CoV, and rSADS‐ΔORF3/GFP. The immunization and challenge protocols were designed based on previously established SADS‐CoV neonatal mouse infection models, with modifications for evaluation of maternally mediated protection [14]. Mice were orally inoculated with 1.5 × 10^6^ TCID_50_ of rSADS‐CoV or rSADS‐ΔORF3/GFP or with an equal volume of DMEM, at 14‐day intervals for three doses. Six dams were included in each group. The serum was collected immediately before the second and third inoculations for measurement of virus‐specific neutralizing antibodies. Females were mated after the second inoculation, and pregnancy, delivery, and nursing were monitored daily.

For offspring challenge, 2‐day‐old suckling pups were allocated to four groups: mock, DMEM, rSADS‐CoV, and rSADS‐ΔORF3/GFP. The age of pups, oral inoculation route, and viral challenge dose were selected based on previously established SADS‐CoV neonatal mouse infection models, which demonstrated that oral administration of SADS‐CoV results in severe disease and viral replication in neonatal mice [14, 15]. Mock pups were born to DMEM‐inoculated dams and were not challenged. Pups in the remaining three groups were orally challenged with 1 × 10^6^ TCID_50_ of rSADS‐CoV. The DMEM group comprised challenged pups born to DMEM‐inoculated dams, whereas the rSADS‐CoV and rSADS‐ΔORF3/GFP groups comprised challenged pups born to dams immunized with the corresponding virus. Pups remained with their biological dams throughout the experiment to preserve maternally mediated protection.

For maternal immunization, the dam was considered the experimental unit. In the offspring challenge experiment, individual pups were challenged and used as measurement units for survival, body‐weight change, tissue viral burden, and histopathological analysis. Because maternal treatment was applied at the dam level, offspring outcomes were interpreted with consideration of potential dam/litter effects. To reduce litter‐associated bias, pups from multiple litters within each treatment group were distributed as evenly as possible for survival monitoring or scheduled tissue collection.

Two independent offspring cohorts were used. In the survival cohort, 12 pups per group were monitored daily for clinical signs, body‐weight change, and survival for 14 days after challenge. Deaths and euthanasia after reaching humane endpoints were recorded as survival events. In the tissue‐collection cohort, 18 pups per group were used for pathological and virological analyses, with six pups per group euthanized at 3, 6, and 10 days postchallenge. At each sampling point, pups were euthanized by carbon dioxide inhalation, and death was confirmed before collection of heart, liver, spleen, lung, kidney, stomach, small intestine, large intestine, and brain. Before brain collection, mice were transcardially perfused with sterile, nuclease‐free PBS to remove the circulating blood. Brain tissues were subsequently excised and thoroughly rinsed with sterile, nuclease‐free PBS before viral RNA extraction or infectious‐virus titration.

Sample size was determined on the basis of previous SADS‐CoV maternal‐protection and suckling‐mouse infection studies, pup availability from each dam, and the need to minimize animal use. No dams or pups were excluded before group allocation. Pups were excluded from scheduled tissue collection only if they died before the planned sampling point or if the collected samples were unsuitable for analysis. The clinical monitoring criteria and humane endpoints were the same as those used in the suckling‐mouse infection experiment. Histopathological and morphometric analyses of intestinal villi and crypts were performed using coded images, where applicable.

### 2.5. RNA Extraction and Viral RNA Quantification

At the indicated time points, tissues from infected mice were homogenized, and total RNA was extracted using the TRIzol reagent according to the manufacturer’s instructions. Viral RNA was quantified by one‐step RT‐qPCR targeting the SADS‐CoV N gene using the HiScript II One‐Step qRT‐PCR Probe Kit (Vazyme, Q222) with gene‐specific primers and probe. Absolute viral RNA copy numbers were determined using a standard curve generated from in vitro‐transcribed RNA containing the target sequence. Based on validation using the RNA standards, samples with a cycle threshold (Ct) value < 35 were considered positive.

For the other RT‐qPCR assays, total RNA was extracted using the TRIzol reagent. cDNA was synthesized using the HiScript III RT SuperMix for qPCR (Vazyme, R323). Quantitative RT‐PCR was performed with the ChamQ Universal SYBR qPCR Master Mix (Vazyme, Q711). The sequences of primers used are listed in Supporting Information 1: Table S1.

### 2.6. Histopathological Analysis

For histopathology, small‐intestinal tissues were collected at 6 days postinfection or 6 days postchallenge as indicated for each experiment. Three pups per group were examined. At the scheduled time point, mice were euthanized by carbon dioxide inhalation under the approved IACUC protocol, and death was confirmed before tissue collection. Tissues were fixed in 4% paraformaldehyde, embedded in paraffin, sectioned, and stained with hematoxylin and eosin. Slides were scanned with a digital slide scanner and assessed for villus shortening, epithelial disruption, mucosal disorganization, and inflammatory‐cell infiltration.

Villus height and crypt depth were measured on H&E‐stained sections, and the villus height‐to‐crypt depth ratio was calculated as an index of intestinal injury. Five to six well‐oriented villus–crypt units were measured per section. Images were coded before the assessment to reduce observer bias. Samples were excluded from morphometric analysis only when tissue orientation or section quality precluded reliable measurement.

### 2.7. Immunofluorescence and Confocal Microscopy

Vero cells were seeded into 6‐well glass‐bottom dishes and infected with rSADS‐ΔORF3/GFP. At 24 h postinfection, cells were permeabilized with PBS containing 1% Triton X‐100 (Diamond, A110694) for 10 min and then blocked with 5% BSA at room temperature for 1 h. Primary antibodies were incubated overnight at 4°C, followed by incubation with fluorophore‐conjugated secondary antibodies for 1 h at 37°C in the dark. Nuclei were counterstained with DAPI (Solarbio, C0065) at room temperature for 10 min. Cells were washed five times with PBS between each step. Images were acquired using a confocal microscope (Leica, Germany) or a super‐resolution imaging system.

### 2.8. Virus Neutralization Assay

Neutralizing antibody titers in dam sera were determined by microneutralization using Vero cells. Sera were heat‐inactivated at 56°C for 30 min and serially diluted twofold in DMEM. Each serum dilution was mixed with an equal volume of rSADS‐CoV containing 100 TCID_50_ and incubated at 37°C for 1 h. The serum–virus mixtures were transferred to confluent Vero cell monolayers in 96‐well plates, with eight replicate wells for each dilution. Virus‐only and cell‐only controls were included in each assay. Cytopathic effect was assessed at 72 h postinfection. The neutralizing titer was calculated by the Reed–Muench method and expressed as the reciprocal serum dilution that prevented the cytopathic effect in 50% of wells.

### 2.9. Western Blotting

For SDS‐PAGE, protein samples were lysed using either cell lysis buffer for Western and IP (Beyotime, P0013) or RIPA lysis buffer (Beyotime, P0013C), both supplemented with an EDTA‐free protease inhibitor cocktail (Roche, 4693132001) and a phosphatase inhibitor cocktail (Beyotime, P1082). Following SDS‐PAGE, proteins were transferred to a nitrocellulose membrane (Merck Millipore, ISEQ00010). The membrane was blocked in tris‐buffered saline with tween (TBST) containing 5% blotting‐grade blocker (Beyotime, P0216) and then incubated with the appropriate primary antibodies, followed by HRP‐conjugated secondary antibodies. Signals were developed using an enhanced chemiluminescence kit (NCM, P10300) and detected with an ECL imaging system.

### 2.10. Statistical Analyses

Statistical analyses were performed in GraphPad Prism v.10.0. Quantitative data are shown as the mean ± s.d. unless stated otherwise. Survival was analyzed using Kaplan–Meier curves and compared by the log‐rank (Mantel–Cox) test. Two‐group comparisons were performed using two‐tailed unpaired Student’s *t*‐tests. Comparisons involving more than two groups or multiple time points were analyzed by one‐way or two‐way ANOVA, followed by the multiple‐comparison test specified in the figure legends. Where applicable, normality and homogeneity of variance were assessed before parametric testing; otherwise, the corresponding nonparametric test was used. For maternal‐protection experiments, offspring‐level outcomes were interpreted with consideration of potential dam/litter effects as maternal treatment was applied at the dam level. *p* < 0.05 was considered statistically significant; significance levels are indicated as  ^∗^ *p* < 0.05,  ^∗∗^ *p* < 0.01, and  ^∗∗∗^ *p* < 0.001.

## 3. Results

### 3.1. ORF3 Depletion Permits Virus Rescue but Reduces Replication in Porcine ST Cells

To determine whether ORF3 is required for SADS‐CoV recovery and replication in vitro, we used the previously established rSADS‐CoV reverse‐genetic system to replace the ORF3 region with GFP and rescued the ORF3‐deficient recombinant virus rSADS‐ΔORF3/GFP (Figure 1A). The recombinant plasmid was cotransfected with pRK5‐N into BHK‐21 cells, and virus‐containing supernatants were subsequently propagated in Vero cells (Figure 1B). Infection of Vero cells with the tenth‐passage virus produced robust GFP fluorescence. Immunofluorescence staining with an antibody against the SADS‐CoV N protein showed that GFP‐positive cells also expressed the N protein, confirming successful rescue of rSADS‐ΔORF3/GFP (Figure 1C).

**Figure 1:**
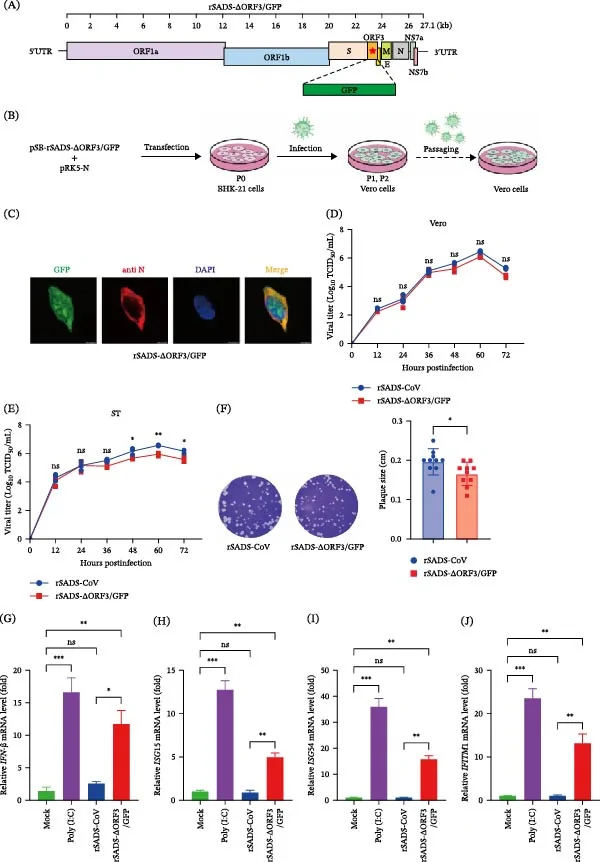
Rescue and in vitro characterization of rSADS‐ΔORF3/GFP. (A) Schematic of the SADS‐CoV CH/GD‐01/2017 genome organization (top) and construction strategy for the ORF3‐deficient recombinant virus rSADS‐ΔORF3/GFP (bottom), in which the ORF3 region was replaced by GFP. (B) Rescue strategy for rSADS‐ΔORF3/GFP. The ORF3‐depletion recombinant plasmid was cotransfected with the helper plasmid pRK5‐N into BHK‐21 cells to generate passage‐0 virus, followed by serial passage in Vero cells. (C) Immunofluorescence analysis of Vero cells infected with the tenth‐passage rSADS‐ΔORF3/GFP. Cells were stained with an antibody against the SADS‐CoV N protein, and colocalization of GFP and N protein was examined by confocal microscopy. (D) Growth kinetics of rSADS‐CoV and rSADS‐ΔORF3/GFP in Vero cells. Cells were infected at a multiplicity of infection (MOI) of 0.01, and viral titers were determined at the indicated time points. (E) Growth kinetics of rSADS‐CoV and rSADS‐ΔORF3/GFP in ST cells. Cells were infected at an MOI of 0.1, and viral titers were determined at the indicated time points. (F) Plaque‐size analysis of rSADS‐CoV and rSADS‐ΔORF3/GFP. Mean plaque diameters were measured for each virus (*n* = 10). (G–J) Relative mRNA levels of *IFN-β* (G), *ISG15* (H), *ISG54* (I), and *IFITM1* (J) in ST cells following infection with rSADS‐CoV or rSADS‐ΔORF3/GFP, as measured by RT‐qPCR.

We next compared the replication kinetics of rSADS‐ΔORF3/GFP and the parental rSADS‐CoV in different cell types. In Vero cells, the two viruses showed similar growth profiles, indicating that the loss of ORF3 did not substantially impair viral replication in this cell line (Figure 1D). By contrast, rSADS‐ΔORF3/GFP replicated less efficiently in ST cells, with significantly lower viral titers at 48 h postinfection (Figure 1E). To determine whether the reduced replication of rSADS‐ΔORF3/GFP in ST cells resulted from defects in the early stages of infection, we compared the attachment and entry efficiency of the two viruses. No significant differences in viral attachment or entry were detected between the two viruses under the conditions tested (Supporting Information 2: Figure S1A,B). These findings suggest that the reduced replication of rSADS‐ΔORF3/GFP in ST cells may involve events after entry, although they do not identify the affected stage. Plaque assays further showed that rSADS‐ΔORF3/GFP formed smaller plaques than those of rSADS‐CoV (Figure 1F). These results indicate that ORF3 is dispensable for SADS‐CoV recovery and replication in Vero cells but contributes to efficient viral replication in ST cells. Given that Vero cells are deficient in type‐I interferon production, we hypothesized that ORF3 may promote SADS‐CoV replication by antagonizing host antiviral signaling in interferon‐competent cells.

To test this possibility, we examined whether the reduced replication of rSADS‐ΔORF3/GFP in ST cells was associated with enhanced antiviral signaling. Following infection with rSADS‐CoV or rSADS‐ΔORF3/GFP, the expression of *IFN-β* and representative *ISGs*, including *ISG15*, *ISG54*, and *IFITM1*, was quantified by RT‐qPCR. Compared with cells infected with parental rSADS‐CoV, rSADS‐ΔORF3/GFP infection elicited markedly stronger induction of *IFN-β* and *ISG* expression (Figure 1G–J). Poly(I:C) increased the expression of the measured antiviral genes, supporting the responsiveness of ST cells in this assay. Together, these findings associate the reduced replication of rSADS‐ΔORF3/GFP with increased *IFN-β* and *ISG* expression but do not establish a direct mechanism of ORF3‐mediated interferon antagonism.

### 3.2. ORF3 Depletion Attenuates SADS‐CoV Pathogenicity in Suckling Mice

To evaluate the contribution of ORF3 to SADS‐CoV pathogenicity in vivo, 2‐day‐old SPF suckling mice were orally inoculated with 1 × 10^6^ TCID_50_ of rSADS‐CoV or rSADS‐ΔORF3/GFP, whereas control mice received culture medium (Figure 2A). Mortality was first observed at 3 days postinfection in both infected groups, indicating that both viruses were able to cause disease in suckling mice. However, during the 14‐day observation period, mice infected with rSADS‐CoV showed significantly lower survival than those infected with rSADS‐ΔORF3/GFP (Figure 2B). Consistent with the survival data, rSADS‐CoV infection markedly impaired body‐weight gain, whereas this effect was less pronounced in the rSADS‐ΔORF3/GFP group (Figure 2C).

**Figure 2:**
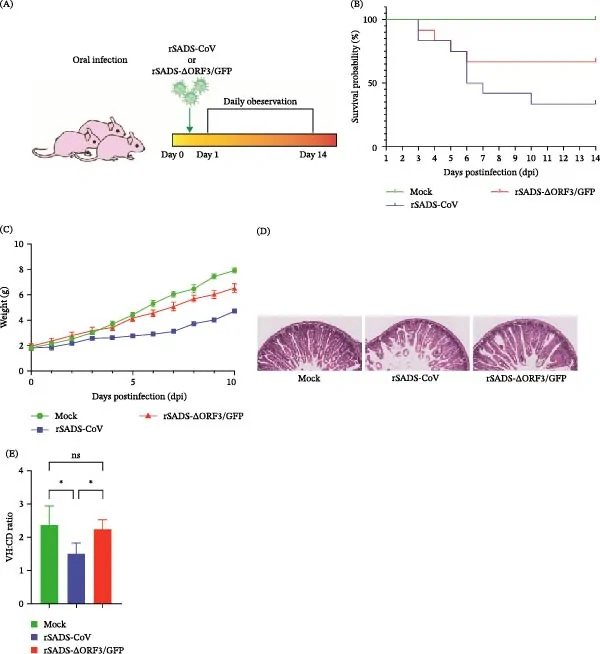
ORF3 depletion attenuates SADS‐CoV pathogenicity in suckling mice. (A) Experimental scheme for infection of suckling BALB/c mice with rSADS‐CoV or rSADS‐ΔORF3/GFP. Two‐day‐old mice were orally inoculated with 1 × 10^6^ TCID_50_ of rSADS‐CoV or rSADS‐ΔORF3/GFP and monitored for clinical outcome and survival for 14 days. Control mice received culture medium. (B) Survival curves of suckling mice orally inoculated with rSADS‐CoV or rSADS‐ΔORF3/GFP. (C) Body‐weight changes in infected suckling mice during the 14‐day observation period. (D) Small‐intestinal sections collected at 6 days postinfection were subjected to H&E staining to assess virus‐induced intestinal pathology. (E) Villus height and crypt depth were measured to calculate the villus height‐to‐crypt depth ratio (VH:CD ratio), which was used to quantify mucosal injury.

Because SADS‐CoV primarily causes enteric disease, we then examined the small‐intestinal pathology after infection. H&E staining showed marked mucosal injury in rSADS‐CoV‐infected mice, with disrupted villi, epithelial damage, and inflammatory‐cell infiltration. These lesions were less severe in mice infected with rSADS‐ΔORF3/GFP, whereas villus architecture was largely preserved in mock‐infected mice (Figure 2D). We then measured the villus height and crypt depth to calculate the VH:CD ratio. The rSADS‐CoV group showed a significantly lower VH:CD ratio than both the mock and rSADS‐ΔORF3/GFP groups, indicating more pronounced villus atrophy and intestinal mucosal injury after parental virus infection (Figure 2E). Together, the lower mortality, improved weight gain, and reduced intestinal pathology observed after rSADS‐ΔORF3/GFP infection indicate that loss of ORF3 attenuates SADS‐CoV pathogenicity in suckling mice and suggest that ORF3 contributes to viral virulence in vivo.

### 3.3. ORF3 Depletion Reduces SADS‐CoV Viral Burden in Multiple Tissues

To determine whether the attenuated phenotype of rSADS‐ΔORF3/GFP was associated with an altered viral burden in vivo, we quantified viral RNA in major organs and intestinal tissues of infected suckling mice. Viral RNA was readily detected in the heart, liver, spleen, lung, kidney, stomach, small intestine, large intestine, and brain after infection with either rSADS‐CoV or rSADS‐ΔORF3/GFP, indicating that ORF3 loss did not prevent systemic tissue distribution. However, ORF3 deletion markedly reduced viral RNA accumulation across tissues. Compared with parental rSADS‐CoV, rSADS‐ΔORF3/GFP showed significantly lower viral RNA loads in all examined organs (Figure 3A–I), with the most pronounced and sustained reductions in the stomach, small intestine, large intestine, and brain (Figure 3F–I). Viral RNA was undetectable in DMEM‐inoculated control mice. To determine whether the reduced viral RNA levels in the brain reflected the presence of infectious virus, we quantified infectious viral titers in brain tissues collected at 1 day postinfection by a TCID_50_ assay. Infectious SADS‐CoV was recovered from these brain samples, and infectious titers were lower in the rSADS‐ΔORF3/GFP group than in the rSADS‐CoV group (Supporting Information 3: Figure S2A). These findings complement the tissue viral RNA measurements but do not establish productive infection of brain parenchymal cells or identify the infected cell types. These data indicate that ORF3 is dispensable for systemic dissemination but supports efficient viral replication in tissues, providing a virological basis for the reduced pathogenicity of rSADS‐ΔORF3/GFP in suckling mice.

**Figure 3:**
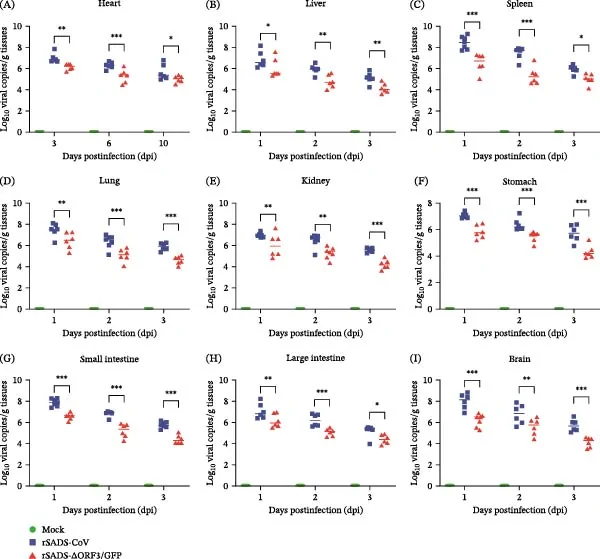
ORF3 loss reduces SADS‐CoV viral burden in multiple tissues. Two‐day‐old BALB/c suckling mice were orally inoculated with rSADS‐CoV or rSADS‐ΔORF3/GFP, and tissues were collected at 3, 6, and 10 days postinfection to assess viral distribution and tissue viral burden in vivo. Heart, liver, spleen, lung, kidney, stomach, small intestine, large intestine, and brain tissues were collected for RNA extraction, and SADS‐CoV viral RNA copies were quantified by RT‐qPCR. Viral loads are shown as viral RNA copies per gram of tissue. (A–I) Viral RNA levels in the heart, liver, spleen, lung, kidney, stomach, small intestine, large intestine, and brain, respectively. Differences in viral burden between infected groups were analyzed at the indicated time points.

### 3.4. ORF3 Depletion Confers Partial Maternally Mediated Protection Against SADS‐CoV Challenge

Maternal immunity is a major source of protection against enteric CoV infection during early life when neonatal immune responses are not yet fully developed. As ORF3 loss reduced SADS‐CoV replication and disease severity in suckling mice, we examined whether it also affected the maternally mediated protection induced by infection. BALB/c female mice were inoculated with rSADS‐CoV, rSADS‐ΔORF3/GFP, or DMEM at 14‐day intervals for a total of three inoculations. Mating occurred after the second inoculation, and the third inoculation was administered before parturition. Offspring were then divided into four groups: an unchallenged mock group and three rSADS‐CoV‐challenged groups born to DMEM‐inoculated, rSADS‐CoV‐inoculated, or rSADS‐ΔORF3/GFP‐inoculated dams. At 2 days of age, pups from the DMEM, rSADS‐CoV, and rSADS‐ΔORF3/GFP inoculation groups were orally challenged with 1 × 10^6^ TCID_50_ of rSADS‐CoV, whereas pups in the mock group were left unchallenged and served as age‐matched, uninfected controls (Figure 4A).

**Figure 4:**
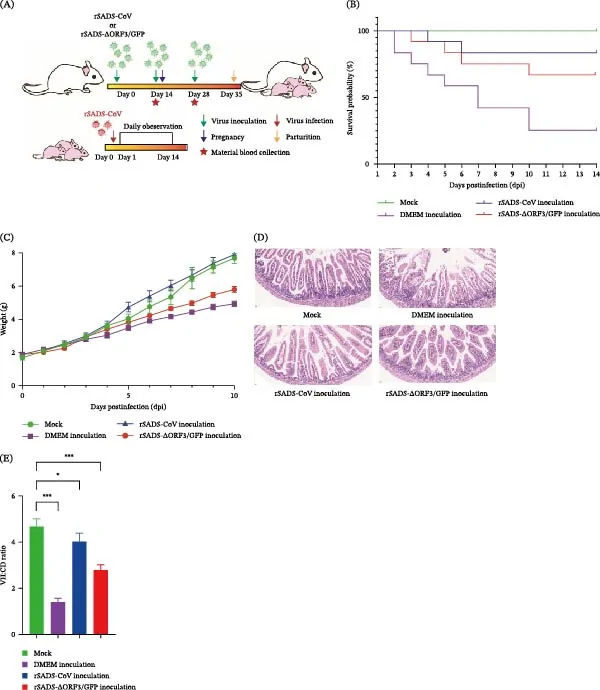
ORF3 depletion confers partial maternally mediated protection against SADS‐CoV challenge. (A) Experimental scheme for maternal immunization and offspring challenge. BALB/c female mice were immunized with rSADS‐CoV or rSADS‐ΔORF3/GFP or inoculated with control medium. Offspring were divided into four groups: an unchallenged mock group and three rSADS‐CoV‐challenged groups derived from DMEM‐inoculated, rSADS‐CoV‐inoculated, or rSADS‐ΔORF3/GFP‐inoculated dams. Two‐day‐old pups in the challenged groups were orally inoculated with 1 × 10^6^ TCID_50_ of rSADS‐CoV and monitored for clinical outcome and survival. (B) Survival curves of pups from the indicated groups. Pups in the mock group were not challenged, whereas pups in the DMEM, rSADS‐CoV, and rSADS‐ΔORF3/GFP groups were orally challenged with rSADS‐CoV. (C) Body‐weight changes of challenged pups during the observation period. (D) H&E staining of small‐intestinal tissues collected at 6 days postchallenge. (E) Villus height and crypt depth were measured to calculate the VH:CD ratio and compare intestinal mucosal injury among the maternal‐protection groups.

After the challenge, pups born to DMEM‐inoculated dams began to die at 2 days postchallenge. Mortality started at day 3 in pups born to rSADS‐ΔORF3/GFP‐inoculated dams and at day 4 in pups born to rSADS‐CoV‐inoculated dams. During the 14‐day observation period, pups from rSADS‐ΔORF3/GFP‐inoculated dams showed lower survival than those from rSADS‐CoV‐inoculated dams, but survival was clearly improved compared with those from DMEM‐inoculated dams. These results indicate that the ORF3‐deficient virus‐induced partial maternally mediated protection (Figure 4B). This difference was also reflected in body‐weight changes. Pups from rSADS‐CoV‐inoculated dams showed only limited impairment of weight gain after the challenge, whereas those from DMEM‐inoculated dams exhibited severe growth retardation. Pups from rSADS‐ΔORF3/GFP‐inoculated dams showed an intermediate level of protection, with greater body‐weight gain than that of the DMEM group but less than that of the rSADS‐CoV group (Figure 4C).

H&E staining further supported this difference in maternally mediated protection. Pups born to DMEM‐inoculated dams and challenged with rSADS‐CoV showed the most severe intestinal injury, characterized by villus shortening, villus disruption, disorganized mucosal architecture, epithelial damage, and marked inflammatory‐cell infiltration. Pups born to rSADS‐ΔORF3/GFP‐inoculated dams showed substantially alleviated intestinal injury compared with pups born to DMEM‐inoculated dams, although their villi remained shorter and less organized than those in pups born to rSADS‐CoV‐inoculated dams. In pups born to rSADS‐CoV‐inoculated dams, the villus structure was largely preserved and resembled that of unchallenged mock controls, with only mild pathological changes (Figure 4D). We further quantified intestinal injury by measuring the villus height and crypt depth and calculating the villus height‐to‐crypt depth ratio. Pups from rSADS‐CoV‐inoculated dams showed the highest VH:CD ratio, whereas pups from DMEM‐inoculated dams showed the lowest ratio. The rSADS‐ΔORF3/GFP group displayed an intermediate phenotype, with a VH:CD ratio lower than that of the parental rSADS‐CoV group but significantly higher than that of the DMEM group (Figure 4E).

Together, these results establish a key feature of ORF3‐depleted SADS‐CoV, which is attenuated yet retains measurable immunogenicity in the maternal‐protection model. Although the protection induced by rSADS‐ΔORF3/GFP was less robust than that of the parental virus, it remained clearly superior to that of the DMEM control. This partial retention of immunogenicity despite attenuation defines the tradeoff that makes rSADS‐ΔORF3/GFP a candidate for LAV.

### 3.5. ORF3 Depletion Reduces Maternal Restriction of SADS‐CoV Burden After Challenge

We next assessed whether the maternal protection induced by rSADS‐ΔORF3/GFP was accompanied by a restriction of viral dissemination after challenge. Viral RNA was quantified in the heart, liver, spleen, lung, kidney, stomach, small intestine, large intestine, and brain of suckling mice at 3, 6, and 10 days postchallenge. In pups from DMEM‐inoculated dams, the rSADS‐CoV challenge resulted in high viral RNA burdens across all examined tissues, indicating extensive systemic and enteric replication in the absence of maternal immunity. By contrast, pups from rSADS‐CoV‐inoculated dams showed a marked reduction in viral RNA loads in all tissues, demonstrating efficient maternally mediated control of viral replication. Pups from rSADS‐ΔORF3/GFP‐inoculated dams also showed reduced tissue viral burdens relative to those of the DMEM group, although this reduction was incomplete. Across most tissues and time points, viral RNA levels in the rSADS‐ΔORF3/GFP group remained higher than those in the rSADS‐CoV group, including in the intestine, stomach, spleen, and brain, where viral replication was consistently detectable (Figure 5A–I). No viral RNA was detected in mock‐treated controls. These data indicate that ORF3‐deficient SADS‐CoV retains the capacity to induce maternal antiviral protection, but this protection is weaker than that elicited by the parental virus, resulting in only partial control of viral replication after challenge.

**Figure 5:**
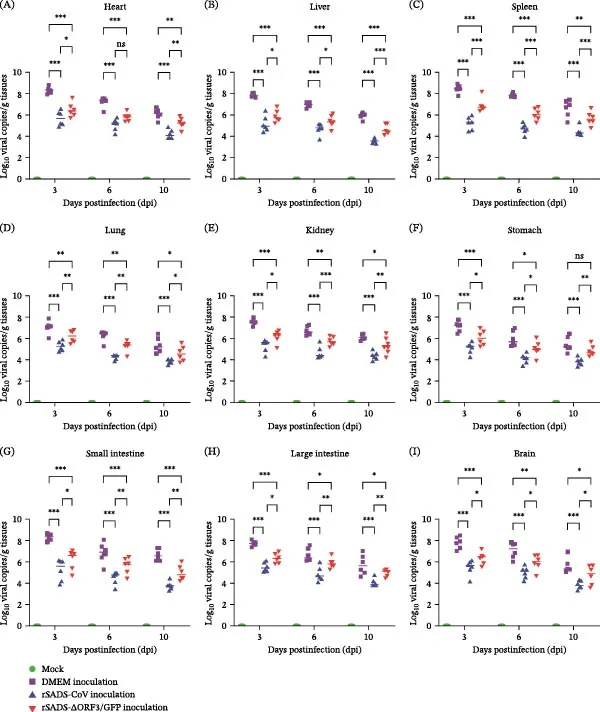
ORF3 loss reduces maternal restriction of SADS‐CoV burden after challenge. BALB/c pups born to dams in the rSADS‐CoV protection, rSADS‐ΔORF3/GFP protection, or control protection groups were orally challenged with rSADS‐CoV at 2 days of age. Heart, liver, spleen, lung, kidney, stomach, small intestine, large intestine, and brain tissues were collected at 3, 6 and 10 days postchallenge to assess tissue distribution and viral burden. RNA was extracted from each tissue, and SADS‐CoV viral RNA copies were quantified by RT‐qPCR and expressed as viral copies per gram of tissue. (A–I) Viral RNA levels in the heart, liver, spleen, lung, kidney, stomach, small intestine, large intestine, and brain, respectively. Differences in viral burden among the indicated protection groups were analyzed at each time point.

### 3.6. ORF3 Depletion Reduces Neutralizing Antibody Responses in Immunized Dams

To explore the basis of the partial protection observed in offspring from rSADS‐ΔORF3/GFP‐inoculated dams, we measured neutralizing antibody responses in sera from inoculated female mice. Serum samples were collected at 14 and 28 days after inoculation and analyzed for 50% neutralization titers. Both rSADS‐CoV and rSADS‐ΔORF3/GFP induced detectable neutralizing antibodies, whereas sera from DMEM‐inoculated mice showed little neutralizing activity. Neutralization titers increased after booster inoculation in both virus‐inoculated groups, indicating that both viruses were immunogenic; however, titers in the rSADS‐ΔORF3/GFP group remained lower than those in the rSADS‐CoV group at both time points (Figure 6A, B). Before assessing the antiviral activity of the sera in cell culture, we examined whether serum treatment itself affected cell viability using the CCK‐8 assay. No significant differences in cell viability were observed among cells treated with sera collected from mice inoculated with DMEM, rSADS‐CoV, or rSADS‐ΔORF3/GFP, indicating that the sera had no detectable cytotoxic effect under the conditions used (Supporting Information 4: Figure S3A). We next tested whether these sera could inhibit SADS‐CoV infection in cells. SADS‐CoV was preincubated with sera from each inoculation group before infection, and viral N‐protein expression was examined by western blotting. Sera from rSADS‐CoV‐inoculated dams markedly reduced SADS‐CoV N protein expression, whereas sera from DMEM‐inoculated mice showed little inhibitory effect. Sera from rSADS‐ΔORF3/GFP‐inoculated dams also reduced viral N protein levels but less efficiently than sera from the rSADS‐CoV group (Figure 6C, D). Immunofluorescence analysis (IFA) showed a similar pattern: SADS‐CoV N‐positive signals were strongly reduced by sera from rSADS‐CoV‐inoculated dams, partially reduced by sera from rSADS‐ΔORF3/GFP‐inoculated dams, and largely unaffected by sera from DMEM‐inoculated dams (Figure 6E, F). To further evaluate the inhibitory effect of maternal sera on viral replication, we quantified intracellular SADS‐CoV RNA levels by RT‐qPCR following serum‐mediated neutralization. Consistent with the reductions in viral N protein expression and N‐positive cells, sera from rSADS‐CoV‐inoculated dams produced the greatest decrease in viral RNA levels, whereas sera from rSADS‐ΔORF3/GFP‐inoculated dams showed an intermediate effect between those of the DMEM control and rSADS‐CoV groups (Figure 6G, H). Together, these results show that rSADS‐ΔORF3/GFP induced lower, but detectable, maternal neutralizing antibody titers than that of parental rSADS‐CoV. The lower antibody responses were accompanied by less robust protection in challenged offspring, although the present study does not establish the causal contribution of maternal viral replication or antibody transfer to this difference.

**Figure 6:**
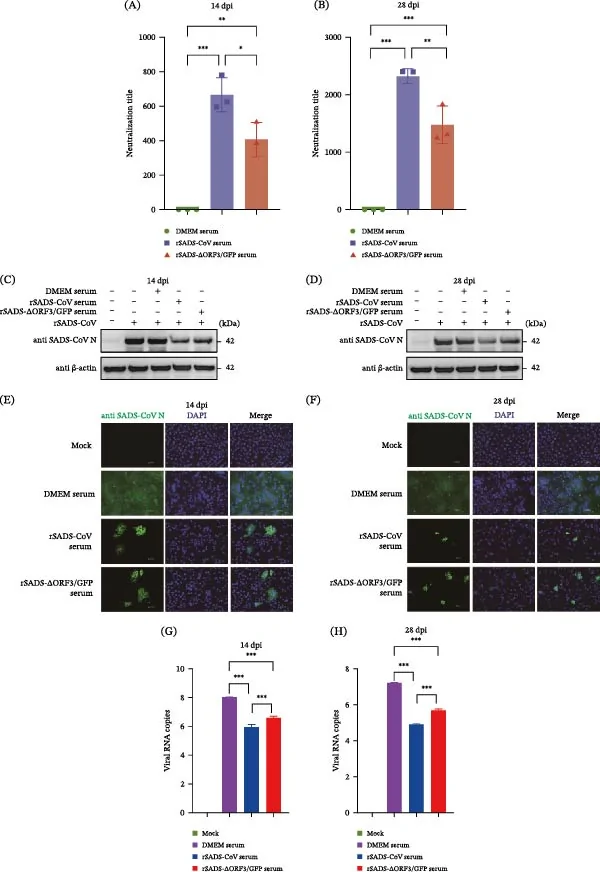
ORF3 loss reduces neutralizing antibody responses in immunized dams. (A, B) Serum samples were collected from female mice at 14 and 28 days after inoculation. Serially diluted sera were incubated with rSADS‐CoV and then added to Vero cells in 96‐well plates. The 50% neutralization titers were calculated by the Reed–Muench method based on the number of wells showing virus‐induced cytopathic effects. (C, D) To assess the inhibitory activity of sera against SADS‐CoV infection, sera from each inoculation group were incubated with rSADS‐CoV at 37°C for 2 h before infection of Vero cells. At 24 h after infection, cell lysates were collected and analyzed by western blotting using antibodies against SADS‐CoV N protein and β‐actin. (E, F) Under the same treatment conditions, Vero cells infected with the serum–virus mixtures were fixed at 24 h after infection and analyzed by immunofluorescence staining with an antibody against SADS‐CoV N protein, followed by an Alexa Fluor 488‐conjugated goat antirabbit secondary antibody. (G, H) Under the same treatment conditions, total RNA was extracted from Vero cells infected with serum–virus mixtures at 24 h after infection, and intracellular SADS‐CoV RNA levels were quantified by RT‐qPCR.

## 4. Discussion

This study links the ORF3‐deficient SADS‐CoV reporter virus to reduced pathogenicity in suckling mice, lower neutralizing antibody responses in inoculated dams, and partial maternally mediated protection in challenged offspring. ORF3 deletion attenuated viral replication and disease severity in suckling mice, but this attenuation was accompanied by lower neutralizing antibody responses in inoculated dams and less robust protection in challenged offspring. These results reveal a tradeoff between virulence and maternal protective efficacy. Nevertheless, the ORF3‐deficient virus retained measurable immunogenicity and conferred clear protection relative to the DMEM control, indicating that attenuation did not abolish its capacity to induce maternally transferred immunity.

Coronavirus accessory proteins are frequently dispensable for virus recovery in cultured cells, yet they often shape viral fitness, host adaptation, and virulence in vivo [31]. Consistent with this view, replacement of SADS‐CoV ORF3 with GFP did not prevent recovery of infectious rSADS‐ΔORF3/GFP, indicating that ORF3 is not essential for the rescue of recombinant virus. ORF3 loss, however, produced a cell‐type‐dependent phenotype: replication was largely preserved in Vero cells but reduced in porcine ST cells, and the mutant virus formed smaller plaques than the parental rSADS‐CoV. Because GFP replaced the ORF3 coding region in rSADS‐ΔORF3/GFP, a potential contribution of reporter‐gene expression to this phenotype should also be considered. Nevertheless, GFP insertion does not necessarily impose a general fitness cost on coronaviruses. In TGEV, another porcine alphacoronavirus, replacement of ORF3A with GFP generated a recombinant virus with growth kinetics comparable to those of the parental virus [32]. Similarly, GFP‐expressing recombinant SARS‐CoV and SARS‐CoV‐2 viruses have shown replication kinetics comparable to those of their corresponding parental or wild‐type viruses in cultured cells [33, 34]. Consistent with these observations, rSADS‐ΔORF3/GFP and parental rSADS‐CoV displayed comparable growth kinetics in Vero cells in the present study, arguing against a generalized, nonspecific impairment of viral replication caused by GFP expression. However, comparable growth in Vero cells does not exclude cell‐type‐specific or in vivo effects of GFP expression. Because the present comparison does not separate ORF3 loss from GFP insertion, the observed phenotype cannot be attributed exclusively to ORF3 deletion.

Similar context‐dependent effects of accessory‐gene deletion have been reported for other coronaviruses. PEDV ORF3 can be deleted or replaced by a reporter gene without abolishing virus propagation, although ORF3 variation affects viral proliferation, cell adaptation, and pathogenicity [27, 35, 36]. Deletion of IBV accessory genes 3a or 3b also attenuates viral phenotypes [26]. These observations suggest that ORF3‐family proteins are not required for basal replication but can support viral fitness in a host‐ or cell‐context‐dependent manner. The differential growth of rSADS‐ΔORF3/GFP in Vero versus ST cells is consistent with this paradigm: Vero cells lack interferon production [37], whereas ST cells possess intact antiviral signaling. We therefore speculate that SADS‐CoV ORF3 may function, at least in part, by counteracting type‐I interferon responses in porcine cells, a hypothesis supported by the known immunomodulatory activities of related ORF3 proteins, as discussed below. This interpretation is consistent with reports that coronavirus ORF3‐related proteins modulate antiviral pathways: SARS‐CoV‐2 ORF3a inhibits Sendai virus‐induced IFN‐β promoter activation [38] and suppresses IFN‐activated JAK/STAT signaling through SOCS1 induction and JAK2 degradation [39]. Similarly, PEDV ORF3 inhibits type‐I interferon signaling by downregulating components of the RLR pathway [18]. Although these observations do not define the mechanism of SADS‐CoV ORF3, they point to *IFN-β* induction, *ISG* expression, and the RIG‐I/MDA5–MAVS–TBK1–IRF3 axis as priority pathways for future study. The increased *IFN-β* and *ISG* expression observed after rSADS‐ΔORF3/GFP infection provides additional evidence of an association between the replication phenotype and host antiviral responses. However, these transcriptional measurements do not establish that altered interferon signaling causes the difference in viral replication.

The impact of ORF3 depletion was more pronounced in vivo than in cultured cells. Relative to the parental rSADS‐CoV, rSADS‐ΔORF3/GFP caused lower mortality, less growth impairment, and milder intestinal injury, with better‐preserved villus architecture and reduced inflammatory damage. These changes coincided with lower viral RNA burdens in multiple tissues, including the intestine and brain. Although RT‐qPCR does not distinguish infectious virus from viral RNA, the reduced tissue burden supports a role for ORF3 in sustaining SADS‐CoV replication or persistence during infection, thereby contributing to tissue damage and disease severity. The contribution of ORF3 to SADS‐CoV pathogenicity is further supported by studies on other swine enteric CoVs. Previous work in piglets associated NS3a deletion with milder pathological changes, although viral shedding and tissue viral loads were not significantly different from those in the wild‐type group [40]. PEDV studies have also linked ORF3 integrity to virulence, as a naturally occurring ORF3‐truncated strain that emerged after serial passage showed reduced pathogenicity in piglets, including improved survival, lower viral shedding, and milder histopathological injury [41]. Likewise, cell‐culture attenuation of SADS‐CoV can markedly alter the disease outcome in piglets [42]. Together, these findings support ORF3 as a determinant of SADS‐CoV fitness and virulence in the suckling‐mouse model while also indicating that the effects of ORF3 loss on disease severity and viral burden may depend on the model and experimental context.

The reduced viral burden and attenuated disease caused by rSADS‐ΔORF3/GFP raised the question of whether attenuation also altered the protection transferred from immunized dams to their offspring. In the maternal‐protection model, offspring from rSADS‐ΔORF3/GFP‐inoculated dams showed delayed disease progression, reduced tissue viral burdens, and attenuated intestinal injury relative to offspring from DMEM‐inoculated dams, indicating that the ORF3‐depleted virus retained detectable protective immunogenicity. This protection, however, was less robust than that induced by parental rSADS‐CoV, consistent with the lower neutralizing antibody titers in inoculated dams and the less efficient restriction of viral replication in challenged offspring. The reduced immunogenicity of the ORF3‐deficient virus likely reflects its attenuated replication, which may limit the antigen load and thereby reduce the magnitude of the neutralizing antibody response in inoculated dams. This association between viral replication capacity and maternal antibody induction highlights the challenge of balancing attenuation with sufficient immunogenicity during LAV development: sufficient attenuation to ensure safety must be balanced against sufficient replication to drive protective antibody titers. This interpretation is consistent with passive‐protection studies of swine enteric CoVs. In PEDV infection, lactogenic antibodies delivered through colostrum and milk are closely associated with protection in neonatal piglets, including reduced disease, lower viral shedding, and improved survival after challenge [28–30]. A previous SADS‐CoV suckling‐mouse study also showed that maternal immunization with SADS‐CoV partially protects offspring, supporting the use of this model to examine maternally mediated protection [14].

The use of suckling BALB/c mice in this setting also extends previous work showing that neonatal mice support age‐dependent SADS‐CoV infection, viral dissemination, and disease and further indicates that this model can be used not only to compare viral virulence but also to preliminarily evaluate maternally mediated protection [14, 15]. Nevertheless, the source and route of protection in our model require further investigation. Because suckling mice continuously receive maternal factors through nursing, milk‐derived antibodies are likely to contribute to protection against intestinal infection [43, 44]. However, maternal antibodies can also be transferred before birth in mice, and this study did not distinguish the relative contributions of prenatal transfer and postnatal lactogenic immunity. Future work should therefore compare SADS‐CoV‐specific IgG, IgA, and neutralizing activity in dam serum, milk, pup serum, and intestinal contents. Together, our data suggest that vaccine strategies capable of inducing strong maternal neutralizing antibody responses may help protect newborn piglets against SADS‐CoV, although this possibility should be tested directly in pregnant sows and their offspring [28, 29, 45].

Several limitations of this study should be considered. Although the suckling‐mouse model enables rapid and cost‐effective comparison of SADS‐CoV virulence and maternally mediated protection, it does not fully recapitulate infection in neonatal piglets, the natural host [3, 14]. The role of ORF3 in virulence, intestinal injury, and maternal protection should therefore be validated in pregnant sows and newborn piglets. In addition, the ORF3‐depletion virus used in this study replaced ORF3 with GFP. An ORF3 revertant or complementation virus would help confirm that the observed changes in replication, pathogenicity, and maternal protection are directly attributable to ORF3 loss. The mechanism by which ORF3 regulates SADS‐CoV fitness and immunogenicity also remains to be defined. Given the reported functions of ORF3‐related proteins in ion‐channel activity, endolysosomal trafficking, viral egress, and immune modulation, future work should examine whether SADS‐CoV ORF3 affects RIG‐I/MDA5–MAVS–TBK1–IRF3 signaling, JAK/STAT activation, lysosomal function, or other membrane‐associated steps of the viral life cycle [19, 39, 46].

Overall, rSADS‐ΔORF3/GFP exhibited reduced pathogenicity while retaining measurable maternal immunogenicity and partial protection of the offspring. These findings highlight a tradeoff between attenuation and maternal protective efficacy in the suckling‐mouse model. We therefore propose rSADS‐ΔORF3/GFP as a candidate LAV backbone that warrants further optimization, including evaluation in pregnant sow models. The contribution of GFP insertion and the relevance of these findings to the natural host remain unresolved.

## 5. Conclusions

In conclusion, the ORF3‐deficient reporter virus rSADS‐ΔORF3/GFP showed reduced pathogenicity in suckling mice while retaining detectable maternal neutralizing antibody responses and partial protection of challenged offspring. These findings support a contribution of ORF3 to SADS‐CoV disease severity and highlight the relationship between attenuation and maternally mediated protective efficacy. However, the effects of ORF3 loss cannot be fully separated from GFP insertion, and the findings should not be directly extrapolated to neonatal piglets.

## Author Contributions

**Nian-Qi Zhang**: writing – original draft, data curation, methodology, conceptualization, formal analysis. **Wei Huang and Zhong-Li Zhang**: investigation, validation, data curation. **Pan-Pan Zhao, Zhao-Yi Xu, Nian-Jin Zhang, and Zhi-Gang Xie**: investigation. **Yong-Le Yang**: investigation, data curation, funding acquisition. **Feng Cong**: supervision, writing – review and editing. **Yao-Wei Huang and Bin Wang**: conceptualization, funding acquisition, supervision, writing – review and editing.

## Funding

This work was supported by the Joint Fund of the National Natural Science Foundation of China (Grants U22A20521, 32302841, and 32302873), the Guangdong Basic and Applied Basic Research Foundation (Grant 2025A1515010899), the Specific University Discipline Construction Project (Grant 2023B10564003), and the Guangdong Laboratory of Lingnan Modern Agriculture Project (Grant NG2022001).

## Disclosure

All authors have read and agreed to the published version of the manuscript.

## Conflicts of Interest

The authors declare no conflicts of interest.

## Supporting Information

Additional supporting information can be found online in the Supporting Information section.

## Supporting information


**Supporting Information 1** Table S1: List of primers and probes used in RT‐qPCR.

**Supporting Information 2** Figure S1: ORF3 deletion does not affect SADS‐CoV adsorption and internalization in ST cells.

**Supporting Information 3** Figure S2: Infectious SADS‐CoV titers in brain tissues of infected suckling mice.

**Supporting Information 4** Figure S3: Serum treatment has no cytotoxic effect on Vero cells.

## Data Availability

The data that support the findings of this study are available in the Supporting Information of this article.
