# Supplementary material for: ORF3 Depletion Attenuates SADS‐CoV Virulence and Retains Partial Maternally Mediated Protection in Suckling Mice
Source: Transbound Emerg Dis. 2026 Oct 1;2026:6713584. doi: 10.1155/tbed/6713584 (PMC13629294; doi:10.1155/tbed/6713584)
Supplement: Supplementary file 1 [file TBED-2026-6713584-s002.pdf]

Figure. S1

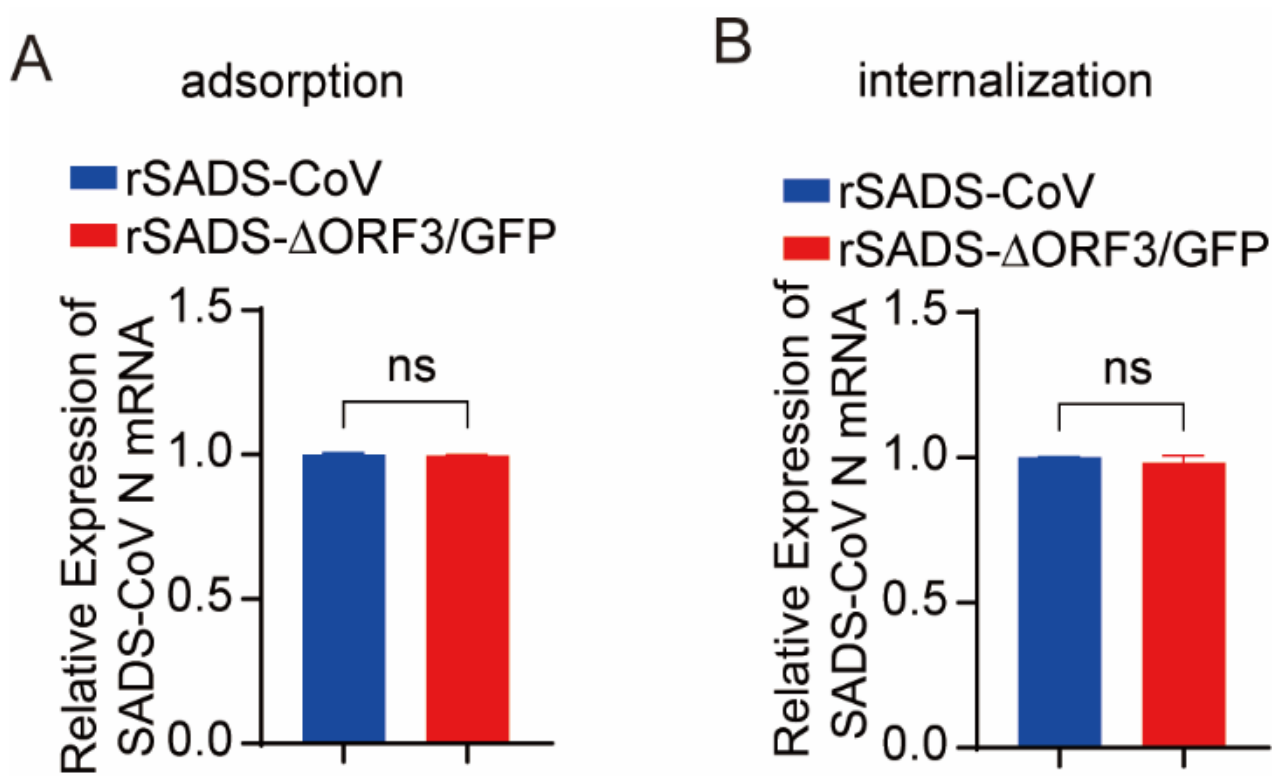

FIGURE S1. ORF3 deletion does not affect SADS-CoV adsorption and internalization in ST cells. (A, B) ST cells were infected with rSADS-CoV or rSADS-ΔORF3/GFP, and viral RNA levels associated with cell adsorption (A) or internalization (B) were quantified by RT-qPCR. No significant differences were observed between the two viruses.

Figure. S2

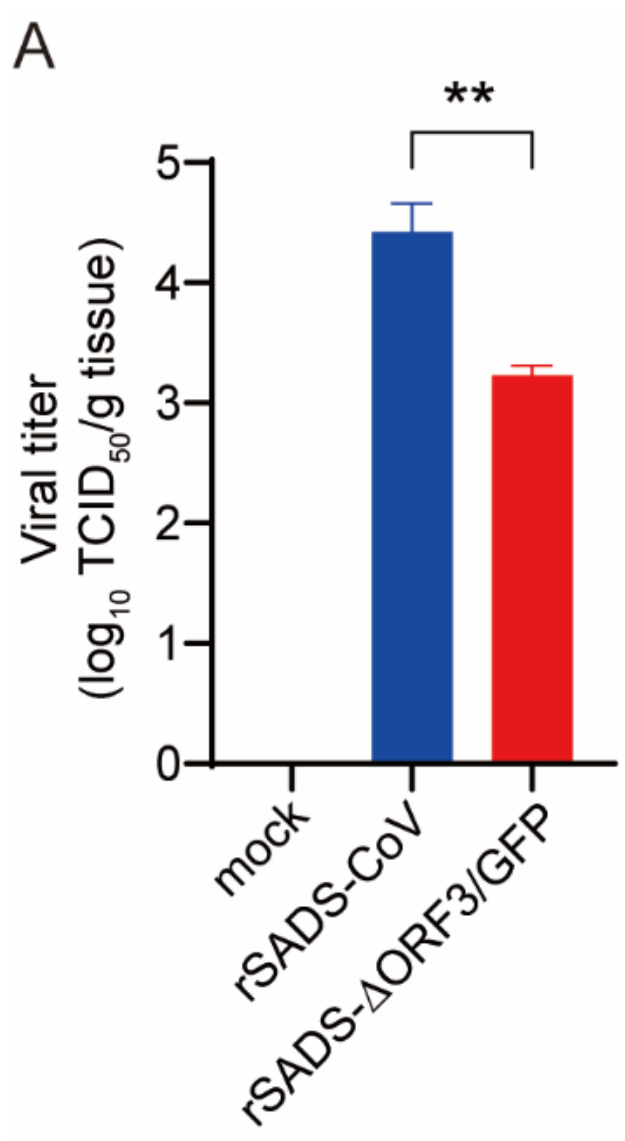

FIGURE S2. Infectious SADS-CoV titers in brain tissues of infected suckling mice. **(A)** Brain tissues were collected from suckling mice infected with rSADS-CoV or rSADS-ΔORF3/GFP at 1 dpi, and infectious viral titers were determined by $TCID_{50}$ assay. rSADS-ΔORF3/GFP-infected mice showed significantly lower infectious viral titers in brain tissues compared with rSADS-CoV-infected mice.

Figure. S3

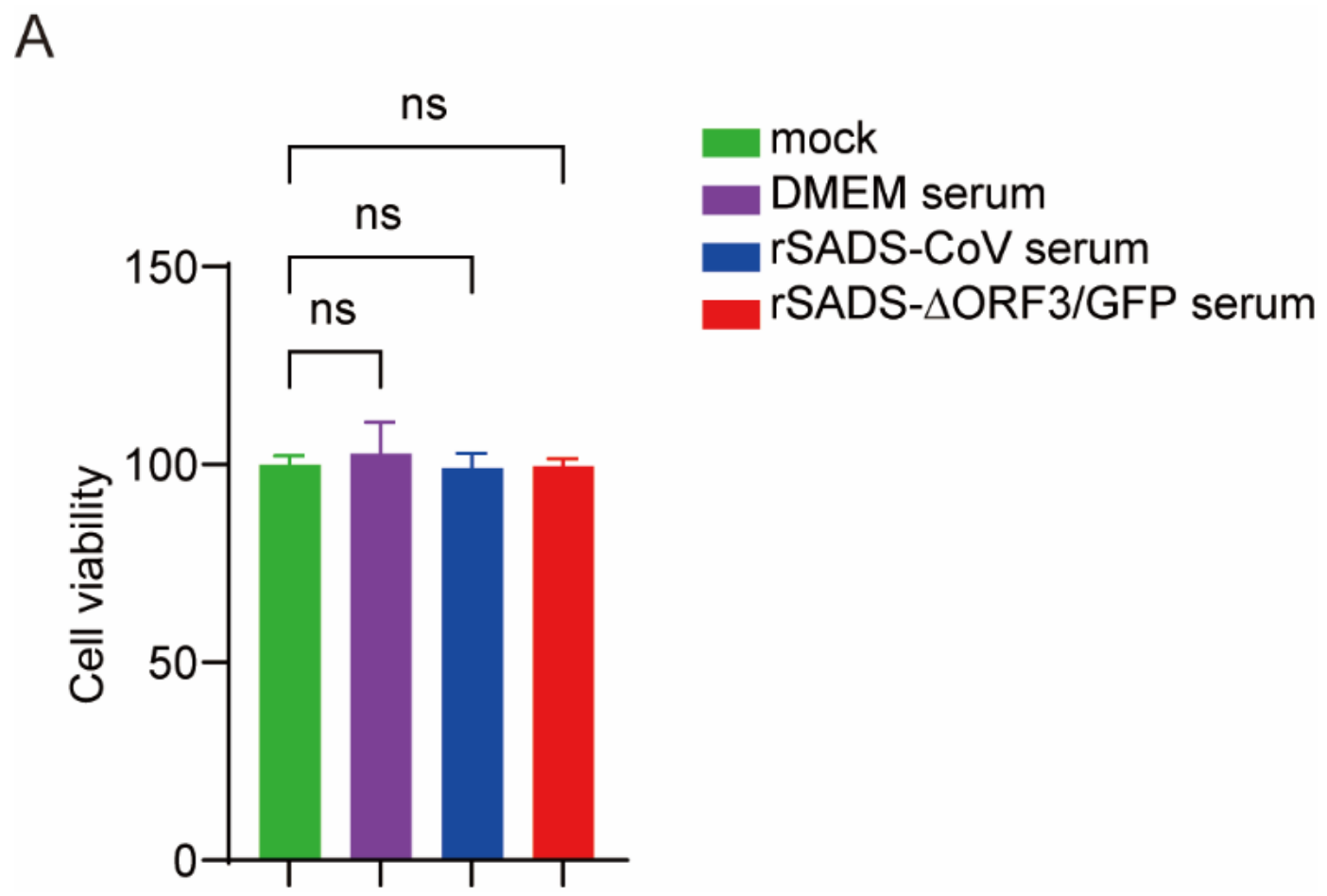

FIGURE S3: Serum treatment has no cytotoxic effect on Vero cells. (A) Vero cells were treated with sera from different inoculation groups, and cell viability was measured by CCK-8 assay. No significant differences were observed among the groups.
