# Supplementary material for: ORF3 Depletion Attenuates SADS‐CoV Virulence and Retains Partial Maternally Mediated Protection in Suckling Mice
Source: Transbound Emerg Dis. 2026 Oct 1;2026:6713584. doi: 10.1155/tbed/6713584 (PMC13629294; doi:10.1155/tbed/6713584)

Figure 6C

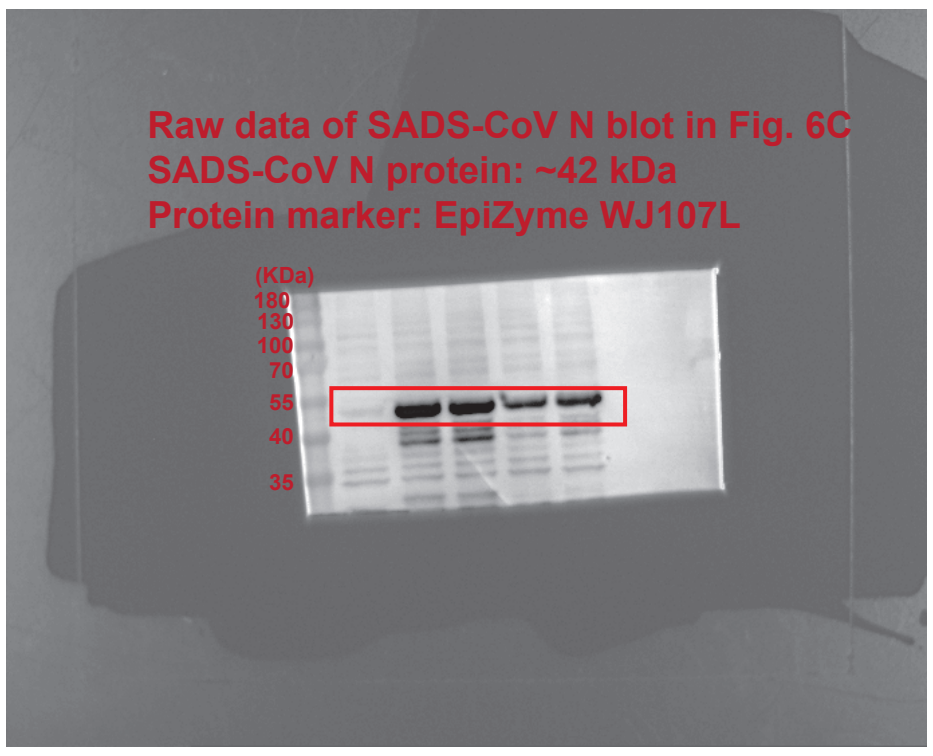

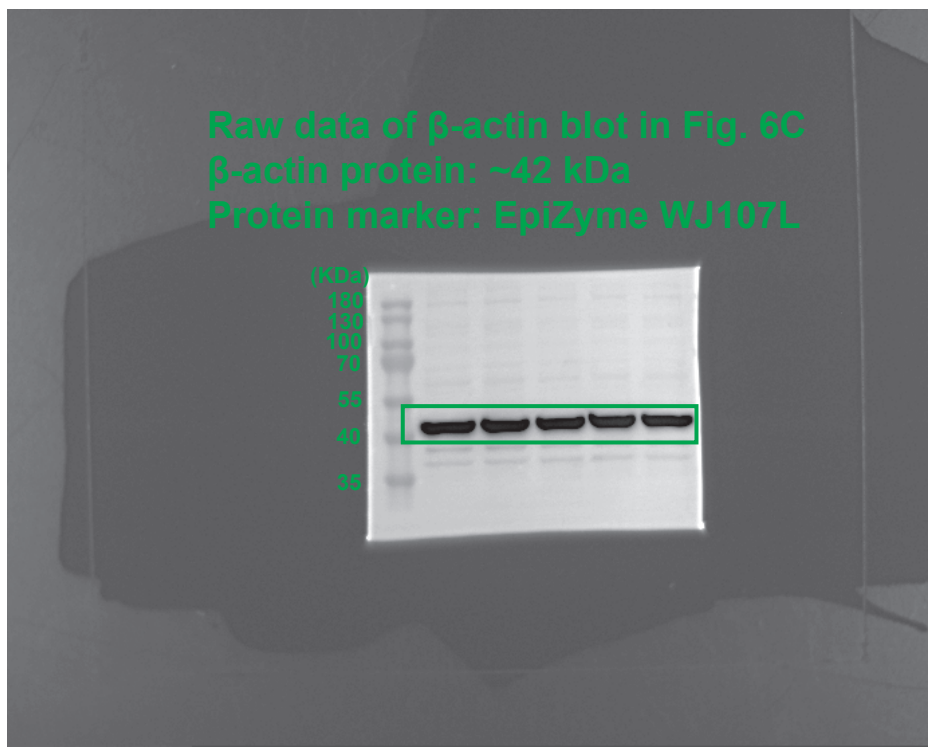

Figure 6D

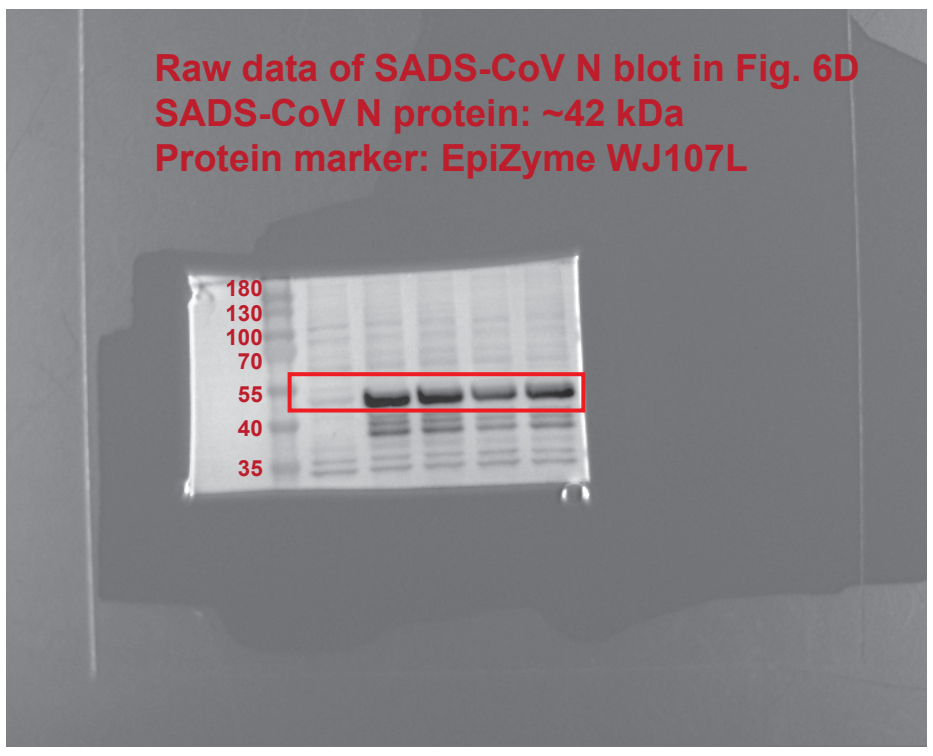

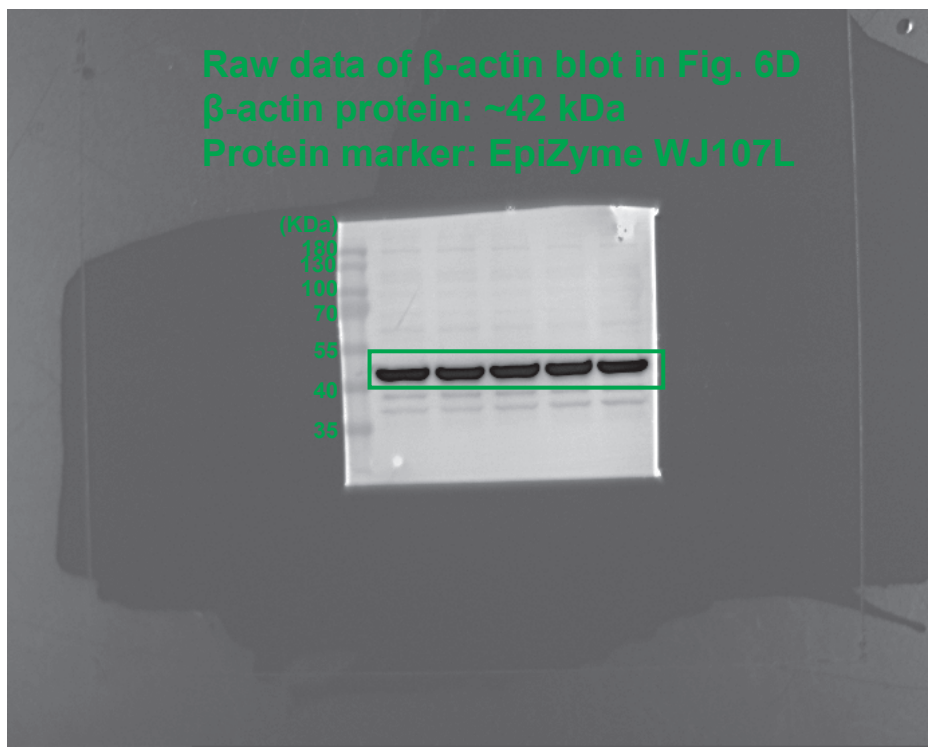

Supplement: Supplementary file 2 [file TBED-2026-6713584-s004.pdf]
