# Supplementary material for: ORF3 Depletion Attenuates SADS‐CoV Virulence and Retains Partial Maternally Mediated Protection in Suckling Mice
Source: Transbound Emerg Dis. 2026 Oct 1;2026:6713584. doi: 10.1155/tbed/6713584 (PMC13629294; doi:10.1155/tbed/6713584)
Supplement: Supplementary file 3 [file TBED-2026-6713584-s003.docx]

**Supplementary Table 1．List of primers and probes used in RT-qPCR.**

| Primer^a^ | Sequence (5’-3’) |
| --- | --- |
| Probe | FAM-TGAAACCCAAACTGAGGTGTAGCAGG-TAMRA |
| SADS-N-F | CTAAAACTAGCCCCCCAGGTC |
| SADS-N-R | TGATTGCGAGAACGAGACTG |
| IFNB-F | AGCACTGGCTGGAATGAAAC |
| IFNB-R | TCCAGGATTGTCTCCAGGT |
| ISG15-F | TGCTGGGAGGCAAGGAGA |
| ISG15-R | CCAGGATGCTCAGTGGGTCT |
| ISG54-F | CTTGTCTGCTCCTATCTCG |
| ISG54-R | CTCCTTTGATTCCTGGTT |
| IFITM1-F | TGCCTCCACCGCCAAGT |
| IFITM1-R | GTGGCTCCGATGGTCAGAAT |

^a^ F, forward primer; R, reverse primer.
